# Unraveling the Relationship Between Trait Self-Control and Subjective Well-Being: The Mediating Role of Four Self-Control Strategies

**DOI:** 10.3389/fpsyg.2019.00706

**Published:** 2019-03-27

**Authors:** Kristian S. Nielsen, Wencke Gwozdz, Denise De Ridder

**Affiliations:** ^1^Department of Management, Society and Communication, Copenhagen Business School, Frederiksberg, Denmark; ^2^Department of Consumer Behaviour, Justus-Liebig-University Giessen, Giessen, Germany; ^3^Department of Social, Health and Organizational Psychology, Utrecht University, Utrecht, Netherlands

**Keywords:** trait self-control, self-control strategies, subjective well-being, structural equation modeling, cross-cultural survey

## Abstract

Although several studies provide evidence that trait self-control contributes to subjective well-being, the self-control strategies that promotes happiness and life satisfaction remains unknown. The present study aims to shed light on this relation by investigating the mediating role of four self-control strategies: situation selection, attentional deployment, reappraisal, and inhibition. To test the hypothesis that self-control strategies mediate trait self-control’s effect on well-being, an online questionnaire on trait self-control, self-control strategies, and cognitive and affective well-being was administered to 4,036 participants from four countries (ages 18–65 and 56.4% female), whose responses were analyzed using structural equation modeling. Our analysis replicates previous studies that trait self-control positively relates to subjective well-being. Moreover, our analysis provides evidence that this relation is indeed mediated by the tendency to employ particular self-control strategies. Attentional deployment and reappraisal positively relate to subjective well-being, whereas inhibition exhibits a negative relation. Situation selection was unrelated to subjective well-being. The incorporation of self-control strategies represents the first attempt to empirically disentangle the positive relation between trait self-control and subjective well-being. The heterogeneous effects of self-control strategies suggest the importance of obtaining a better understanding of which aspects of trait self-control positively contributes to subjective well-being.

## Introduction

Abundant evidence is accumulating on high self-control’s wide-reaching benefits, ranging from enhanced academic performance to stronger interpersonal relationships to better mental and physical health (Tangney et al., 2004; De Ridder et al., 2012). Low self-control, in contrast, is associated with numerous negative personal and societal outcomes, including obesity, criminality, substance abuse, and procrastination (Gottfredson and Hirschi, 1990; Patton et al., 1995; Baumeister and Heatherton, 1996). These observations give good reason to expect a positive relation between trait self-control and subjective well-being, which has also been found in recent studies (e.g., Cheung et al., 2014; Hofmann et al., 2014; De Ridder and Gillebaart, 2017; Wiese et al., 2017).

One of the first studies to empirically test the relation between trait self-control and subjective well-being is Hofmann et al. (2014). In three studies, the authors find that trait self-control is positively correlated with both affective and cognitive well-being. Specifically, high trait self-control is linked to greater levels of cognitive well-being and positive affect and less frequent experiences of negative affect. Whereas Hofmann et al. (2014) suggest that the effect is mediated by affective experiences, other studies that validate this positive relationship propose regulatory focus (Higgins, 1997) as mediator of the relation (Cheung et al., 2014). But importantly, the specific aspects of trait self-control that positively contribute to subjective well-being have yet to be uncovered.

In the process of unraveling trait self-control’s relationship with subjective well-being, we adapt the widely recognized conceptualization of subjective well-being as consisting of two distinct components: affective well-being and cognitive well-being (Diener, 1984; Luhmann et al., 2012b). Affective well-being refers to the frequency and intensity of positive and negative emotions and mood (Luhmann et al., 2012a), whereas cognitive well-being entails the cognitive evaluation of people’s overall satisfaction with life (Diener et al., 2010a).

The positive association between trait self-control and subjective well-being is particularly intriguing because it challenges the stereotypical perception of high self-control as a dutiful self-discipline in which individuals deny themselves (short-term) joys and pleasure (Hofmann et al., 2014; Wirtz et al., 2016). It thus raises the question of which aspects of trait self-control are actually making people happier and more satisfied with their lives. Whereas the exercise of self-control has traditionally been interpreted as a process of overriding or inhibiting unwanted impulses that may interfere with long-term goal striving (Baumeister et al., 1998), several scholars now suggest that the concept should be broadened to encompass strategies other than effortful inhibition (e.g., Fujita, 2011; Hofmann and Kotabe, 2012; Gillebaart and de Ridder, 2015; Duckworth et al., 2016; Hoyle and Davisson, 2016). These additional self-control strategies include forming goal-congruent habits (De Ridder et al., 2012; Adriaanse et al., 2014; Galla and Duckworth, 2015) and using goal support from others (Nielsen and Bauer, 2018). In relation to subjective well-being, De Ridder and Gillebaart (2017) argue that individuals with high trait self-control report higher subjective well-being not because they are more competent in inhibiting short-term temptations but because they are better at initiating goal-directed behaviors.

To account for other self-control strategies that supplement effortful inhibition, some scholars propose an altered definition of self-control as “the process or behavior of overcoming a temptation or prepotent response in favor of a competing goal” (Milyavskaya et al., 2019). This definition better allows for the integration of different regulation strategies, some of which are derived from the related fields of self-regulation, desire regulation, emotion regulation, and mood regulation. Hence, to better understand the underlying mechanisms of self-control’s contribution to subjective well-being, we explore the role of effortful inhibition and three other self-control strategies.

## Self-Control Strategies

Our investigation focuses on four self-control strategies: *situation selection*, *attentional deployment*, *reappraisal*, and *inhibition*. These strategies draw inspiration from recent theoretical research on self-control strategies, including the process model of self-control (Duckworth et al., 2016). The process model of self-control stipulates that desires develop in an iterative process beginning with the situation and ending with a response tendency. This model builds on Gross’s (1998) well-established process model of emotion regulation, and outlines the cyclic stages in which desires evolve and are amplified or weakened over time (Duckworth et al., 2016). Specifically, we adopt the process model’s hierarchical categorization of self-control strategies to support our aim of providing evidence that a tendency to use particular strategies can advance our understanding of the relation between self-control and well-being.

Recent research has proposed that self-control is most effective when exerted as early in the process as possible (Fujita, 2011; Hofmann and Kotabe, 2012; Mann et al., 2013; Gillebaart et al., 2016). For example, individuals at a pub with friends who see other customers smoking may themselves experience the desire for a cigarette. Although this scenario does not automatically represent a self-control dilemma, it can do so for individuals who are trying to break the smoking habit. Although the first and most proactive approach is to avoid such a tempting situation (situation selection) in favor of others that support long-term goals, once the desire has arisen, the second approach is to shift attention away from the problematic situation (attentional deployment) and toward non-tempting stimuli or thoughts such as thinking about the next holiday destination. The third and fourth approaches are to alter the meaning of the cigarette (reappraisal) – for example, to a source of bad smelling clothes and hair or cause of cancer – or simply inhibit the desire to smoke (inhibition). Each of these strategies is further detailed in the discussion below:

*Situation selection* plays on the unique human ability to imagine and forecast future events, including their consequences for affect, motivation, and cognition (Gilbert and Wilson, 2007). By employing this capability, individuals can identify future situations that might elicit tempting desires. The most effective self-control strategy relies on this prospective ability and involves the selection and prioritization of situations that support long-term goals and restrict the availability of such desires (Aspinwall and Taylor, 1997). Situation selection thus refers to approaching or avoiding certain situations, places, people, or objects in order to shield and advance important long-term goals (Gross, 1998). Recent evidence suggests that trait self-control is associated with the employment of this kind of strategies, as is for example demonstrated with more frequent use of proactive strategies (Hofmann et al., 2012a; Ent et al., 2015; Gillebaart et al., 2016) and earlier detection of self-control conflicts. Although an effective strategy for all individuals, this strategy may be especially beneficial for individuals with limited capabilities in later stages of the self-control cycle, who can use it to reduce their likelihood of self-control failures. On the other hand, because the complexity and unpredictability of everyday life may sometimes make the strategy infeasible, it cannot be the only means of effective self-control and should be supplemented with other strategies.

One such alternative is *attentional deployment*, which allows individuals to voluntarily focus or shift attention elsewhere in situations that cannot be changed or escaped (Eisenberg et al., 2001). In this strategy, attentional processes are used to direct attention away from tempting stimuli and facilitate the refocusing of attention on neutral and non-tempting stimuli or thoughts (Eisenberg et al., 2016). Two attentional deployment techniques (Gross, 1998) are particularly relevant for self-control: distraction, the selective focusing of attention on a specific situational aspect or shifting attention away from the situation altogether (Mallory and Rupp, 2016); and concentration, the ability to actively focus on specific tasks. In a situation of temptation, distraction may entail focusing on other objects or events in the physical environment or redirecting attention inwards to non-tempting memories or images (Gross and Thompson, 2007). Consistent with the elaborated intrusion theory of desire (Kavanagh et al., 2005), distraction should be engaged in as early in the process as possible to constrain the development and elaboration of a tempting desire (Hofmann et al., 2012b). Concentration, on the other hand, involves focusing on tasks that promote long-term goals while blocking intrusive thoughts of proximal desires. This strategy, being highly adaptive in a self-control context (Shah et al., 2002), tends to be frequently employed from infancy to adulthood when other more prospective strategies are impossible or unsuccessful (Magen and Gross, 2010).

When paying attention to tempting stimuli is unavoidable, *reappraisal* can diminish the strength of tempting desires and amplify the strength of desires congruent with long-term goals (Ochsner and Gross, 2005). Reappraisal involves the use of mental strategies to alter perceptions of an object, behavior, situation, or feeling (Mischel et al., 1972; Gross, 1998) either prior to, during, or after an event (see Quoidbach et al., 2015, for an overview). Put simply, reappraisal entails thinking about something in a different way that favors a person’s long-term goals. In general, converging evidence indicates that reappraisal can strongly impact affective reactions to tempting stimuli and provide an effective means of down regulating desires (e.g., Hofmann et al., 2010). Moreover, although reappraisal is a valuable self-control strategy, it is mainly effective for low to modest levels of affective intensity. When applied under very high affective intensity, its effect seems to break down (Sheppes et al., 2009; Gross, 2015), indicating that in these situations, other self-control strategies should take precedence.

The most studied strategy in the self-control research is *inhibition*, which refers to the process of inhibiting pre-potent thoughts, feelings, or behavioral tendencies and refraining from acting on them (Tangney et al., 2004; Vohs et al., 2008). Individuals call upon inhibition when the experience of a tempting desire triggers a pre-potent action tendency. If unattended, the tempting desire will be enacted, leading to self-control failure. Because the purpose of inhibition is to prevent the action tendency from influencing behavior until the desire episode fades out, it is deemed to be necessary when other self-control strategies have been unsuccessful. It is also considered the last stage in the self-control cycle (Mann et al., 2013), primarily because of its reliance on an effortful allocation of both cognitive and motivational resources. Here, the cognitive component is the inhibitory control capacity, which is strongly dependent on dispositional (e.g., working memory capacity) and situational (e.g., cognitive capacity) factors (Friese et al., 2009), while the motivational component is the motivation to recruit these inhibitory capacities when available, a concept that lies at the core of research on ego depletion and willpower (Baumeister et al., 1998, 2007; Inzlicht and Schmeichel, 2012; Friese et al., 2018). This prerequisite of both cognitive and motivational resources, however, makes inhibition a difficult undertaking, one whose effectiveness can be expected to fluctuate considerably.

## Self-Control and Subjective Well-Being

Not only does previous research document the positive correlation between trait self-control and subjective well-being (e.g., Cheung et al., 2014; Hofmann et al., 2014), it also adds important nuances to the conventional view of high self-control as stifled by dutiful self-discipline and a blatant defiance of pleasurable experiences. Among these is its broadened focus on the features of trait self-control that actually make people happier and more satisfied with their lives. For example, Hofmann et al. (2014) attribute the positive relation to more adept goal balancing and less frequent experiences of goal conflict in individuals with high trait self-control. De Ridder and Gillebaart (2017), on the other hand, credit a better initiation of goal-directed behavior rather than a competent inhibition of short-term temptations. Given inhibition’s greater error proneness relative to more prospective strategies, both these explanations are hard to reconcile with its role as major driver of the positive relation (Fujita, 2011; Hofmann et al., 2012a). Rather, other self-control strategies may be more effective in reducing goal conflicts and inducing subjective well-being. For instance, the process model of self-control (Duckworth et al., 2016) suggests that early intervention strategies (e.g., situation selection) are preferable to late intervention strategies (e.g., inhibition) in facilitating effective self-control. Such strategies, by hindering temptation development and making resistance less effortful (Kotabe and Hofmann, 2015), facilitate self-control and promote goal progress, which is positively linked to positive affect and subjective well-being (Klug and Maier, 2015; Carver and Scheier, 2016). Restraining the potency of temptations may also help reduce the potentially negative affective impact of resisting temptation (Kavanagh et al., 2005).

Based on the above, we predict that the early stage intervention strategies of situation selection and attentional deployment will be more positively linked to subjective well-being than the later stage strategies of reappraisal and inhibition. In particular, we expect inhibition to exhibit a negative relation to subjective well-being because of the affective costs of inhibiting fully developed temptations and its more unstable effectiveness in facilitating goal progress. While we expect the four strategies to mediate the relationship between trait self-control and subjective well-being, a full mediation is unlikely to be observed as other unassessed strategies also exist (e.g., habit formation, implementation intentions, and goal support).

## Materials and Methods

The data used in this study were part of a larger survey assessing environmentally friendly consumer behavior. Survey responses were collected in Germany, Poland, Sweden, and the United States using an online questionnaire that included numerous measures of psychological constructs and consumer behavior (see Gwozdz et al., 2017; Nielsen and Bauer, 2018, for further details). Because of its breadth, the survey was divided in two parts completed within a 2–4 weeks interval. All measurements discussed here were included in survey part II.

### Procedures

The questionnaire, administered by the market research company Qualtrics between October 2016 and January 2017, was developed in English and subsequently translated into German, Polish, and Swedish by certified ISO17100 translators. All participants were incentivized by points redeemable for different products (e.g., gift cards). To maximize data quality, the questionnaire incorporated several quality measures used to screen out careless responses (Meade and Craig, 2011; DeSimone et al., 2015). The quality measures included instructed items (e.g., “Please select strongly agree”), bogus items (e.g., “I always sleep less than 1 h per night”), checks for answering in patterns (i.e., straight lining), and self-reported data on answer quality (e.g., “In your honest opinion, should we use your data in our analysis of this study”). Respondents who failed instructed items were screened out automatically, while those who failed multiple quality checks were replaced.

### Participants

The target group for the questionnaire was individuals aged 18–65 years. Although the sample for Part I of the survey was representative of the population with regard to age, gender, region, and education (*N* = 10,363), because participants themselves decided whether or not to return for Part II, the process was subject to a self-selection bias and full representativeness unachievable. 4,591 respondents filled in Part I and Part II. Due to missing values in the variables employed – mainly in the self-control strategies (missing values *n* = 555) – in our models, we ended up with a final sample of 4,036 respondents with the following breakout by country: Germany (*n* = 1,059), Poland (*n* = 972), Sweden *(n* = 1,028), and the United States (*n* = 977). We decided to delete the cases with missing values as IBM SPSS AMOS 25.0 (and especially the bootstrapping procedure) cannot handle missing values. The demographic profile of the deleted cases is similar to the remaining cases, thus there was not a systematic bias in who was excluded (descriptive statistics of deleted cases are available from the authors upon request). The mean age of the entire sample was 42.66 (*SD* = 13.53), with 56.4% being female.

### Measures

#### Trait Self-Control

Our measure for trait self-control was the well-validated Trait Self-Control Scale (Tangney et al., 2004), whose 13 items were answered on a 7-point Likert scale (1 = *not at all* to 7 = *very much*) indicating general self-control tendencies; for example, “I am good at resisting temptation,” “People would say that I have iron self-discipline,” and “I am able to work effectively toward long-term goals.” Cronbach’s alpha for this trait self-control scale was 0.85.

#### Situation Selection

We quantified the ability to select situations that favor long-term goals and avoid tempting desires using the scale developed by Ent et al. (2015), which comprise the following 5 items: ”I avoid situations in which I might be tempted to act immorally,” “I choose friends who keep me on track to accomplishing my long-term goals,” “When I work or study, I deliberately seek out a place with no distractions,” “In my life, the line between right and wrong is very clear and sharply drawn,” and “When I want something, I work out a systematic plan for how to get it.” Participants answered on a 7-point Likert scale (1 = *not at all* to 7 = *very much*). Cronbach’s alpha for this situation selection scale was 0.68.

#### Attentional Deployment

To measure attentional deployment, we used the Attention Control subscale of the Adult Temperament Questionnaire – Short Form (Evans and Rothbart, 2007), in which attention control is part of the broader measure of effortful control but specifically measures effortful attention (i.e., the capacity to intentionally focus or shift attention). This scale thus encompasses both distraction and concentration. Participants rated all 5 items on a 7-point Likert scale (1 = *extremely untrue of me* to 7 = *extremely true of me*), including “When I am trying to focus my attention, I am easily distracted,” “It is hard for me to focus my attention when I am distressed,” and “It’s often hard for me to alternate between two tasks.” Cronbach’s alpha for this scale was 0.75.

#### Reappraisal

We assessed this strategy using the Reappraisal Scale from Gross and John’s (2003) well-validated Emotion Regulation Questionnaire (ERQ), which although focused on emotion regulation, has also been used to assess the cognitive reappraisal of desire-related objects, situations, and behaviors (e.g., Giuliani et al., 2013). This scale consists of 6 items measured on a 7-point Likert scale (1 = *strongly disagree* to 7 = *strongly agree*), including “I control my emotions by changing the way I think about the situation I’m in,” “When I want to feel more positive emotion, I change the way I’m thinking about the situation,” and “When I’m faced with a stressful situation, I make myself think about it in a way that helps me stay calm.” Cronbach’s alpha was 0.85.

#### Inhibition

Our measure of inhibition was the Inhibitory Control subscale of the Adult Temperament Questionnaire – Short Form (Evans and Rothbart, 2007), which assesses the ability to bear down positively toned impulses and withstand approach tendencies. All 7 items were measured on a 7-point Likert scale (1 = *extremely untrue of me* to 7 = *extremely true of me*), including “It is easy for me to inhibit fun behavior that would be inappropriate” and “When I see an attractive item in a store, it’s usually very hard for me to resist buying it” (reverse coded). Cronbach’s alpha was 0.53.

#### Affective Well-Being

We assessed affective well-being based on the Scale of Positive and Negative Experience (SPANE) (Diener et al., 2010b), whose 12 items are evenly devoted to positive and negative experiences (6 items each). Although all items are scored on a scale from 1 (*very rarely or never*) to 5 (*very often or always*), the positive and negative scales are scored separately because of the distinction and partial independence of the two types of feelings. The summed positive score (SPANE-P) can range from 6 to 30 with a Cronbach’s alpha of 0.91, while the negative scale (SPANE-N) has the same range but a Cronbach’s alpha of 0.87. The two measures can be combined by subtracting the negative score from the positive score to give SPANE-B scores ranging from -24 to 24.

#### Cognitive Well-Being

Our measure of cognitive well-being was the mean score over all 5 items of the widely-used Satisfaction with Life Scale (Diener et al., 1985), which is designed to assess the cognitive aspects of subjective well-being (e.g., “in most ways my life is close to ideal”). Scored on a 7-point scale from 1 (*strongly disagree*) to 7 (*strongly agree*), the scale had a Cronbach’s alpha of 0.90.

#### Subjective Well-Being

To measure subjective well-being, we created a composite measure of affective and cognitive well-being by applying a confirmatory factor analysis to the measure items for both components. All factor loadings were around 0.70, the average variance explained (AVE) was 0.50, composite reliability (CR) was 0.73 and Cronbach’s alpha was 0.71.

#### Control Variables

Our control variables were age, measured in years; sex, a binary variable equal to 1 if female; country, denoted by a dummy variable for each of the four countries; and income, measured as net income in 11 comparable categories based on national statistics (Eurostat for Germany, Poland and Sweden; U.S. Census Bureau for the U.S.). The income calculation algorithm, using the 2014 statistic for the monthly net income of the 18–64 age group in each country, ensured cross-country comparability through employing a four-step process: (1) identifying the median income per country and using this as the lower boundary of the middle-income category; (2) defining the upper boundary of the lowest category as the poverty line for singles (i.e., 60% of the median income of a single household); (3) defining the lower boundary of the upper level as approximately 2.5 times the median income; and (4) spreading the intervals for the 11 categories evenly.

### Analytic Strategy

To analyze our hypothesized model in which trait self-control was the exogenous variable and the four self-control strategies as well as subjective well-being (affective and cognitive well-being) the endogenous variable, we applied structural equation modeling (SEM) with a maximum likelihood estimator to our calculated scores. All calculations were performed on IBM^®^ SPSS^®^ Amos 25.0, which we also used to estimate the measurement model. Our structural model mirrored the hypothesized relation between trait self-control and subjective well-being both directly and mediated by the four self-control strategies. Testing for mediation, we used the Preacher and Hayes’s (2008) bootstrapping approach in which mediation was accepted if the indirect paths were statistically significant (based on the bootstrapped standard errors). We redrew 2,000 samples for the bootstrapping. We accounted for the nested data structure (i.e., individual respondents within countries) by using a multi-group comparison model, but we did not adjust for multiple testing.

We estimated our structural model three times with varying subjective well-being variables, including a composite measure of subjective well-being as the dependent variable (Model 1), cognitive well-being (Model 2), and affective well-being (Model 3). The overall model fit for all three models met the criteria proposed by Hair et al. (2010): the comparative fit index (CFI), the normed fit index (NFI), and the adjusted goodness of fit (AGFI) were all around 0.94, with a 0.03 root mean square error of approximation (RMSEA).

## Results

Table 1, which reports the descriptive statistics for all measures and the zero-order correlations among them, reveals positive correlations between trait self-control and all four strategies, with correlation coefficients that varied from *r* = 0.15 for reappraisal to *r* = 0.54 for attentional deployment (all at *p* < 0.001). The correlations between trait self-control and the composite measures for subjective well-being were *r* = 0.26 for cognitive well-being and *r* = 0.39 for affective and subjective well-being (all at *p* < 0.001). Affective and cognitive well-being correlated highly with each other (*r* = 0.64, *p* < 0.001). Table 1 also shows positive correlations between the self-control strategies and the various well-being measures, with a range from *r* = 0.07 (inhibition and cognitive well-being) to *r* = 0.34 (attentional deployment and affective well-being). All these correlations were statistically significant.

**Table 1:** Descriptive statistics and correlations.

#	Variable	*M*	*SD*	Min	Max	1	2	3	4	5	6	7	8
1	Trait self-control	4.44	1.01	1	7	–							
2	Situation selection	4.89	1.03	1	7	0.30^∗∗∗^	–						
3	Attentional deployment	4.45	1.21	1	7	0.54^∗∗∗^	0.04^∗∗∗^	–					
4	Reappraisal	4.71	1.13	1	7	0.15^∗∗∗^	0.45^∗∗∗^	0.05^∗∗∗^	–				
5	Inhibition	4.50	0.90	1	7	0.46^∗∗∗^	0.14^∗∗∗^	0.44^∗∗∗^	0.11^∗∗∗^	–			
6	SWB	5.22	1.01	1.60	7.56	0.39^∗∗∗^	0.23^∗∗∗^	0.33^∗∗∗^	0.14^∗∗∗^	0.31^∗∗∗^	–		
7	CWB	4.28	1.48	1	7	0.26^∗∗∗^	0.17^∗∗∗^	0.19^∗∗∗^	0.07^∗∗∗^	0.17^∗∗∗^	0.76^∗∗∗^	–	
8	AWB	6.45	8.12	-24	24	0.39^∗∗∗^	0.17^∗∗∗^	0.34^∗∗∗^	0.16^∗∗∗^	0.25^∗∗∗^	0.96^∗∗∗^	0.64^∗∗∗^	–

*^∗^p < 0.05, ^∗∗^p < 0.01, ^∗∗∗^p < 0.001.*

**FIGURE 1:**
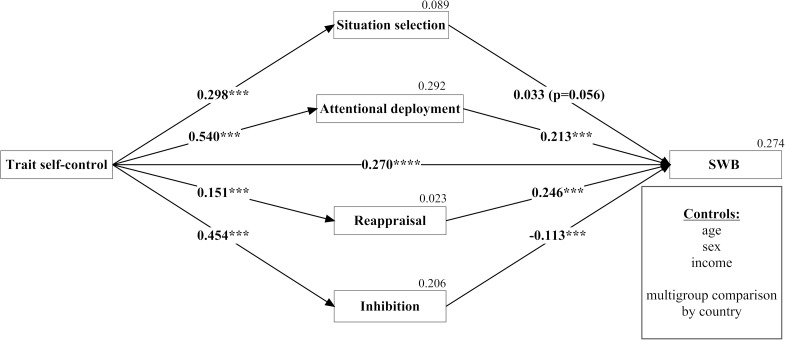
Mediation analysis of the effect of trait self-control on subjective well-being mediated through four self-control strategies. ^∗^*p* < 0.05, ^∗∗^*p* < 0.01, ^∗∗∗^*p* < 0.001; standardixed coefficients, bootstrapped standard n=2,000, multiple squared correlations for dependent variables.

Figure 1 outlines the SEM results for Model 1 in which subjective well-being was the endogenous variable. Although all strategies were statistically significantly associated with trait self-control, the paths were stronger between trait self-control and attentional deployment (β = 0.54, *p* < 0.001) and inhibition (β = 0.45, *p* < 0.001) than between trait self-control and situation selection (β = 0.30, *p* < 0.001) and reappraisal (β = 0.15, *p* < 0.001). This stronger association was confirmed by the 95% confidence intervals reported in Table 2, column 1, which did not overlap with those for situation selection and reappraisal. The associations between trait self-control and the strategies were naturally the same for all three well-being models (note that we cannot infer causality in these associations due to the study’s cross-sectional design).

**Table 2:** The relation between trait self-control (direct and indirect effects), self-control strategies, and subjective well-being.

From	To	(1) SWB^1^	(2) CWB^2^	(3) AWB^3^
Trait self-control	Situation selection	0.298^∗∗∗^		
		[0.268, 0.330]		
	Attentional deployment	0.540^∗∗∗^		
		[0.516, 0.563]		
	Reappraisal	0.151^∗∗∗^		
		[0.113, 0.185]		
	Inhibition	0.454^∗∗∗^		
		[0.427, 0.480]		
Situation selection	SWB/CWB/AWB	0.033	0.042^∗^	-0.003
		[-0.001, 0.067]	[0.006, 0.078]	[-0.039, 0.033]
Attentional deployment	SWB/CWB/AWB	0.213^∗∗∗^	0.100^∗∗∗^	0.202^∗∗∗^
		[0.178, 0.250]	[0.062, 0.138]	[0.166, 0.240]
Reappraisal	SWB/CWB/AWB	0.246^∗∗∗^	0.111^∗∗∗^	0.208^∗∗∗^
		[0.214, 0.278]	[0.075, 0.146]	[0.174, 0.242]
Inhibition	SWB/CWB/AWB	-0.113^∗∗∗^	-0.083^∗∗∗^	-0.087^∗∗∗^
		[-0.146, -0.081]	[-0.118, -0.047]	[-0.121, -0.054]
Trait self-control (direct effect)	SWB/CWB/AWB	0.270^∗∗∗^	0.211^∗∗∗^	0.269^∗∗∗^
		[0.236, 0.308]	[0.171, 0.252]	[0.233, 0.306]
Trait self-control (indirect effect)	SWB/CWB/AWB	0.111^∗∗∗^	0.045^∗∗∗^	0.100^∗∗∗^
		[0.083, 0.136]	[0.019, 0.072]	[0.072, 0.124]

Obs.		4,036	4,036	4,036

Model fit:				
^1^ X^2^= 663.26; *df* = 70; *p* = 0.000; X^2^/*df* = 9.475; CFI = 0.954; AGFI = 0.943; NFI = 0.943; RMSEA = 0.032				
^2^ X^2^= 663.26; *df* = 70; *p* = 0.000; X^2^/*df* = 9.475; CFI = 0.948; AGFI = 0.943; NFI = 0.943; RMSEA = 0.032				
^3^ X^2^= 663.26; *df* = 70; *p* = 0.000; X^2^/*df* = 9.475; CFI = 0.952; AGFI = 0.943; NFI = 0.947; RMSEA = 0.032				

*Standardized coefficients; bias-corrected bootstrapped standard errors. n = 2,000; bootstrapped 95% confidence intervals in parentheses; controls: age, income, sex, and multi-group comparison by country to account for nested data structure; ^∗^p < 0.05, ^∗∗^p < 0.01,^∗∗∗^p < 0.001.*

In addition to demonstrating a positive direct effect between trait self-control and subjective well-being (β = 0.27, *p* < 0.001), we also found that of the four strategies, situation selection was least associated with subjective well-being (β = 0.03, *p* = 0.056), followed by inhibition, which was negatively related (β = -0.113, *p* < 0.001). We observed the strongest positive associations with subjective well-being for attentional deployment (β = 0.21, *p* < 0.001) and reappraisal (β = 0.25, *p* < 0.001). Not only was the indirect effect of trait self-control through mediation of the strategies positive and statistically significant (β = 0.11, *p* < 0.001), but including the strategies as mediators partially explained the total relation between trait self-control and subjective well-being. This total effect (i.e., the sum of the direct and indirect effect) was thus also positive and relatively strong (β = 0.38, *p* < 0.001).

One interesting result of calculating separate structural models for cognitive and affective well-being (Table 2, columns 2 and 3, respectively) was that attentional deployment and reappraisal seemed to be more strongly related to affective well-being than to cognitive well-being. For example, the path coefficient of attentional deployment on cognitive well-being was β = 0.10 (*p* < 0.001), whereas that on affective well-being was β = 0.21 (*p* < 0.001) with no overlapping 95% confidence intervals. Moreover, although the direct effects of trait self-control on both subjective well-being measures could be the same (because the 95% confidence intervals overlap), trait self-control had a stronger indirect effect on affective well-being (β = 0.10, *p* < 0.001) than on cognitive well-being (β = 0.05, *p* < 0.001). This finding could suggest that the strategies played a larger mediating role between trait self-control and affective well-being than between trait self-control and cognitive well-being.

## Discussion

Given the growing attention in recent years to self-control strategies as important explanatory factors of trait self-control’s influence over myriad positive outcomes, including cognitive and affective well-being, this present research examines these strategies in the hope of increasing comprehension of the now firmly established association between trait self-control and well-being. More specifically, because the disconnect between self-discipline and pleasurable experience (e.g., happiness) makes it difficult to understand how self-control driven solely by inhibition positively relates to subjective well-being (Cheung et al., 2014; Hofmann et al., 2014; De Ridder and Gillebaart, 2017), we focus on four strategies recently suggested to be essential for understanding self-control’s link to desired outcomes. By incorporating the strategies of situation selection, attentional deployment, reappraisal, and inhibition, we are able to better determine the specific contribution of self-control’s inhibitive aspect in relation to other strategies. Prior to our analysis, we predicted three outcomes: replication of the earlier research finding that trait self-control is associated with cognitive and affective well-being; self-control strategies would mediate the effect of trait self-control on subjective well-being; and evidence that the early stage strategies of situation selection and attentional deployment are more likely to account for positive effects on well-being.

Our findings lend partial support to these predictions. First, our analysis yields path coefficients of similar magnitude to those of Cheung et al. (2014) and Hofmann et al. (2014), supporting trait self-control’s direct contribution to both the cognitive and affective components of subjective well-being. In reporting these results, we focus on our general model, which indexes both cognitive and affective well-being, because our separate analyses for these two variables revealed no notable differences (although the coefficients of attentional deployment and reappraisal were slightly lower for cognitive than for affective well-being). By incorporating several self-control strategies, we provide empirical evidence for partial mediation; that is, trait self-control is associated with all four strategies, with medium to strong relations for situation selection, attentional deployment, and inhibition and a slightly lower relation for reappraisal. In turn, all strategies except situation selection are related to subjective well-being, with attentional deployment and reappraisal associated with greater well-being but inhibition having a negative relation. Subjective well-being thus seems most strongly associated with attentional deployment and reappraisal. When individuals rely on inhibition as their primary self-control strategy, in contrast, their subjective well-being appears to suffer.

Our findings show that it is not only trait self-control *per se* that is responsible for subjective well-being but also the tendency to use particular strategies that accompany high trait self-control. The expected strong and positive associations between early-stage strategies and subjective well-being are, however, not supported by the results. Particularly, the null-finding between situation selection and subjective well-being contrasts our prediction. This null-finding primarily leaves two possibilities: (1) our way of measuring situation selection was unsuccessful in fully capturing the essence of the strategy; or (2) that the current theorizing around situation selection needs to be revised – at least insofar well-being, instead of other effects, are involved. Future research is encouraged to provide further clarity on the feasibility of either possibilities.

We demonstrate, as predicted, that attentional deployment is significant for subjective well-being, lending some credence to the idea that restraining the potency of temptations can contribute to better well-being outcomes and should be preferred over late stage strategies. Similarly, and in line with our prediction, inhibition is found negatively associated with well-being, no matter whether cognitive or affective. Although supporting our prediction, this observation should be interpreted with some caution due to the low reliability of the inhibition measure, thus restricting our ability to draw strong conclusions about inhibition’s effect on subjective well-being. Inhibition also displays a positive bivariate correlation with subjective well-being—likely suggesting a suppression effect emerging from the introduction of other self-control strategies in the model.

Our study follows a very recent stream of studies showing that trait self-control is associated with a variety of self-control strategies (e.g., Hennecke et al., 2018). This relation is specifically alluded to in the literature aimed at advancing our understanding of self-control beyond effortful inhibition, which challenges the classic definition of self-control in terms of the self’s capacity to override or inhibit undesired inner responses and behavioral tendencies, and to refrain from acting on them (Baumeister et al., 1998; Tangney et al., 2004). In general, these theoretical papers emphasize the strategic nature of self-control, suggesting that as long as reactions are initiated by a self-control dilemma, self-control can take many forms beyond effortfully controlled processes, including the avoidance of tempting situations or the formation of adaptive routines (Fujita, 2011; Hofmann and Kotabe, 2012; Gillebaart and de Ridder, 2015).

Our results suggest that rather than being exclusively or more strongly related to inhibition, trait self-control is also associated with such strategies as situation selection and attentional deployment, albeit rather weakly with reappraisal. This weak association, however, may result from our measure’s strong focus on emotional reappraisal (see Gross and John, 2003), which might insufficiently capture the essence of experiencing a self-control dilemma. Future research might thus examine whether a reappraisal assessment that is more geared toward this dilemma can establish stronger relations with self-control.

Showing that trait self-control relates to other strategies than inhibition, and that the four strategies partially mediate the relationship between trait self-control and subjective well-being, is the most important result of our study. That being said, it should be acknowledged that the direct path between trait self-control and subjective well-being has a stronger effect than the indirect path through the four strategies. The stronger direct effect indicates that while the four strategies account for parts of the relationship, much has yet to be understood in terms of which mechanisms drive the positive relation between trait self-control and subjective well-being.

## Limitations and Future Research

Despite its valuable contribution, our study has several limitations. Our study’s cross-sectional design implies that we cannot infer any causality in the model and are unable to test the superiority of early stage strategies implied by the hierarchy of self-control strategies assumed in our hypotheses (cf. Duckworth et al., 2016). Our use of trait measures that report participant tendencies to employ rather than actual use of the self-control strategies renders an analysis of the immediate affective consequences induced from the use of the strategies impossible.

Another limitation is the low reliability of the inhibition measurement, which prevents us from drawing strong conclusions about inhibition’s relation to subjective well-being. There may also be a non-ideal match between our conceptualization of the self-control strategies and the measurements thereof. Namely, because we decided to adopt existing scales that were not necessarily developed for exact purpose (but previously showed high construct validity), there is a risk that they do not fully correspond to what was conceptually intended. For example, our situation-selection measurement may not capture the full essence of the strategy. As a result, we cannot exclude the possibility that certain elements of situation selection may be associated – either positively or negatively – with subjective well-being.

It is likewise important to note that the survey data has been subjected to multiple testing, as other manuscripts have been published from the same data set (although not with any of the variables included here). Moreover, our hypotheses were not pre-registered. We also recognize the limitations of relying on self-reported strategy use, as people may not be unaware of their strategy use and the self-report could create problems of social desirability, memory bias, and reporting bias. With these limitations in mind, and despite a large cross-country sample, our results should predominantly be regarded as suggestive evidence of the role of self-control strategies in explaining the positive relationship between trait self-control and subjective well-being.

Future research can address many of the shortcomings of our study by employing a longitudinal design. A longitudinal design allows for an assessment of people’s actual employment of the self-control strategies including the affective implications of using either of the strategies (e.g., feelings of guilt or pride), a test of the hierarchical and temporal viewpoint of the strategies, and a generally stronger test of mediation. Future research may also develop more precise measurements of the self-control strategies that more strongly correspond to the conceptualization in recent theoretical research, including the process model of self-control. For example, the current situation-selection measure has low face validity and may be improved by developing items that specifically assess the deliberate preference for situations that promote goal-directed behavior and subjective well-being. Future research may also seek to further develop the conceptualization of situation selection and empirically validate its uniqueness from other situational strategies, such as situation modification (cf. Duckworth et al., 2016).

Most importantly, future research may further investigate which strategies and components of trait self-control are driving its positive relation to subjective well-being. These may include habitualizing goal-directed behavior, using implementation intentions, or engaging in mental contrasting. Future research may, similarly, investigate the existence of other, non-strategy-related mediators, such as goal attainment, cognitive effort in solving self-control dilemmas, or adept goal setting (potentially coupled with affective forecasting).

## Data Availability

The datasets for this study will not be made publicly available because the funding project has a strict policy against publishing the dataset online. However, the dataset can be made available to reviewers upon request.

## Ethics Statement

A full review and ethical approval were not required according to Copenhagen Business School’s guidelines and national regulations. This research was carried out in accordance with the ethical standards of American Psychological Association. The participants signed an informed consent form stating the expected duration of the study and explaining they could withdraw from the study at any point, and that the data would be anonymous.

## Author Contributions

KN, WG, and DD designed the study described in the manuscript and wrote the manuscript. KN and WG collected the data. WG performed the data analyses.

## Conflict of Interest Statement

The authors declare that the research was conducted in the absence of any commercial or financial relationships that could be construed as a potential conflict of interest.
